# Horse Activity Participants’ Perceptions About Practices Undertaken at Activity Venues, and Horse Welfare and Wellbeing

**DOI:** 10.3390/ani15152182

**Published:** 2025-07-24

**Authors:** Julie M. Fiedler, Sarah Rosanowski, Margaret L. Ayre, Josh D. Slater

**Affiliations:** 1 Melbourne Veterinary School, Faculty of Science, University of Melbourne, 250 Princes Highway, Werribee, Melbourne, VIC 3030, Australia; jmfiedler@student.unimelb.edu.au (J.M.F.); jdslater@unimelb.edu.au (J.D.S.); 2 School of Agriculture, Food and Ecosystem Sciences, University of Melbourne, Royal Parade, Parkville, Melbourne, VIC 3010, Australia; mayre@unimelb.edu.au

**Keywords:** equine, horse, equestrian, animal welfare, animal wellbeing, horse racing, animal management practices

## Abstract

Participation in horse-related activities frequently involves relocating horses from the home stable to an activity venue, which might require local, regional, or international travel. In these circumstances, horses are exposed to unfamiliar surroundings and experience changes to their daily routines, which could have negative welfare impacts. An online survey was conducted in 2021 to ask experienced horse sector participants about the horse management practices that they perceived worked well and provided for positive horse welfare when undertaken at venues. Qualitative analysis identified four themes: ‘managing venues’, ‘monitoring fitness to participate’, ‘maintaining a healthy equine digestive system’, and ‘using horse behaviors to inform decision-making’. The findings indicate that activity-related individuals selected practices that assisted horses to adapt to venue surroundings, remain calm, and stay healthy. The co-authors propose that experienced participants recognize that practices include both provisions (inputs) and outcomes (the horse’s subjective experiences), resonating with the Five Freedoms and Five Domains models. For horse activity organizations proposing to implement the Five Domains model, the findings indicate that reviewing practices and implementing updates is timely and achievable. The authors propose that continuously updating practices will contribute to safeguarding horses and maintaining the sector’s social license to operate.

## Simple Summary

The horse sector is highly mobile, involving the regional and international movement of horses for a range of competition activities. Horses are relocated to activity venues and exposed to unfamiliar surroundings and changes to their daily routines, which could have negative welfare impacts. Using an online survey, this study asked experienced horse sector participants about the horse management practices they perceived worked well to promote positive horse welfare at activity venues. Qualitative analysis identified four themes: ‘managing venues’, ‘monitoring fitness to participate’, ‘maintaining a healthy equine digestive system’, and ‘using horse behaviors to inform decision-making’. The results showed that activity participants selected practices that assisted horses to adapt to the venue surroundings, remain calm, and stay healthy. The co-authors propose that participants consider practices both in terms of management inputs, aligning with the Five Freedoms model, and welfare outcomes, resonating with the Five Domains model. For horse activity organizations proposing to implement the Five Domains model, the findings indicate that reviewing current practices and implementing updates to align with the Five Domains model should be achievable. Updating practices in this way will contribute to the safeguarding of horses and to maintaining the sector’s social license to operate.

## 1. Introduction

The global horse industry relies on frequent regional, national, and international movement of horses for competition, recreational activities, breeding, and trade [1]. Horses are regularly relocated from the home stable to activity venues so humans can participate in organized competitive or non-competitive activities [2]. This involves managing horses in surroundings and situations that are unfamiliar to them and disruptive to their daily routine, which can adversely impact their welfare and wellbeing [3,4]. Activity participants expect horses to remain calm and responsive to training cues, be safe to handle, and be fit to participate at the time required by the human’s scheduled activity commitments [5,6,7]. These commitments need to be balanced with the horse’s welfare and wellbeing requirements [7,8].

Practices related to the management, riding, and driving of horses for activities like horse racing or equestrian sports continue a more than 4000-year-old tradition of humans interacting with horses [9]. This study focused on the practices, defined as habitual patterns of actions that occur among like-minded individuals [10,11], undertaken by activity participants, officials, venue managers, and others in the context of organized horse-related activities. While some ancient and traditional practices have been preserved for their cultural significance, such as equitation in the French tradition [11,12], others have been informed by the establishment of equestrian-influenced veterinary schools in the 1700s, the veterinary profession, and the rapidly expanding field of animal welfare science [13,14]. Contemporary animal management practices recognize animal sentience and holistically consider horses’ physical, mental, and social requirements, which involves making inferences about equine mental experiences [15,16]. However, how individuals undertake horse management practices differs due to geographic, cultural, and religious influences, personal worldviews about sentient animals and animal welfare, and ethical perspectives regarding the context and purpose of human interactions with animals [17,18,19,20].

Participation in activities often involves relocating the horse to a venue where the activity occurs for periods of a few hours or up to several weeks [1,2]. Horse carers (alternatively, caretakers or caregivers) need to be cognizant of the potential welfare impact of removing horses from their familiar home stable environment with its predictable routines. For equine welfare and wellbeing to be maintained at the activity venue, carers need to use behavioral interpretations to make inferences about how the horse may be feeling [4,16]. An example are horse behaviors that may occur in response to elevated noise at the venue, which horse carers might infer as anxiety or stress [3,4,21]. Behaviors indicative of stress, such as heightened reactivity, require attention because these present a safety issue for humans and horses, and may predispose horses to a range of health problems, such as colic [4,22,23]. To assist horses in habituating to new environments and reduce stress, horse carers make use of knowledge gained from their personal experience of interacting with horses, animal welfare science, and ethology, and apply this to the selection and implementation of practices [4,24,25].

The extent to which horse management practices provide beneficial welfare outcomes for horses is dependent on factors such as the horse carer’s level of competency and attributes like empathy, which can reduce the likelihood of practices resulting in negative equine mental experiences occurring [26,27,28]. Horse-related activities are, by definition, human-centric scenarios, which means that organizational rules, performance commitments, and activity context increase the complexity of providing for the horses’ physical, mental, and social requirements [29,30,31].

Contemporary horse welfare and wellbeing are assessed using animal-centric tools, which recognize the subjective experiences of animals. The welfare practices employed by those caring for horses can be assessed by their inputs, such as provision of food and water, as articulated by the Five Freedoms model, a framework that guides animal carers about what to do for animals [32]. The Five Freedoms state that animals should have ‘freedom from’ (1) thirst, hunger, and malnutrition, (2) thermal and physical discomfort, (3) pain, injury, and disease, and (4) fear and distress, and (5) ‘freedom to’ express normal behavior [33]. Aligning with each freedom statement is a ‘provision’ statement to inform animal carers’ practices, such as the provision to prevent or rapidly diagnose and provide treatment to keep animals free from pain, injury, and disease [32,33].

Welfare practices can also be assessed by their outcomes, such as the animal’s subjective mental experiences, assessed by the Five Domains model using indicators listed under the domains of physical environment, health, nutrition, behavioral interactions, and mental state [26]. To support decision-making and to improve horse welfare and wellbeing, organizations, including the Fédération Equestre International (FEI) and the International Federation of Horseracing Authorities (IFHA), have adopted the Five Domains model [26,34,35].

Conducting horse-related activities at venues requires organization managers and participants to reconcile providing for horse welfare with a range of constraints, including environmental conditions and facility design [36,37]. For example, organizers and participants need to weigh the trade-offs between the benefits of keeping horses apart at the activity venue to maintain biosecurity with the resultant cost to the horse of negative mental experiences resulting from restricted social interaction [26,36,38]. Careful selection of management practices can partially offset the adverse effects of separated housing conditions by providing environmental and social conditions enriched with opportunities for horses to make meaningful choices when interacting with their surroundings [16,39,40].

Previous research has considered management practices undertaken at the home stable (the horse’s usual residence, incorporating stables, paddocks, and/or yards). Studies about the management practices of Australian horse carers found that most horses were checked by a carer at least once a day, a minority of horses lived permanently in stables, and most horses only received veterinary attention when injured or ill [41,42]. These circumstances differ from those associated with many horse-related activities, where horses are confined, often in stables, checked frequently throughout the day by carers and officials, and undergo mandatory and voluntary examinations by appointed veterinarians [7,43,44]. However, research about management practices undertaken at organized horse sport and leisure activities that also considers participant viewpoints about horse welfare and wellbeing is limited [7]. Whether at the activity venue or the home stable, all decision-makers and individuals in proximity to horses have an ethical and social responsibility to adopt active, collective models of care, referred to in this study as safeguarding [19,45]. This includes a collective responsibility to be aware that an individual’s decisions and actions contribute to the horse’s mental experiences [16,26].

This study aimed to gain insight into the perceptions of experienced horse sector participants about horse-related practices that, when undertaken at activity venues, were believed to work well and benefited animal welfare and wellbeing. The results showed that respondents recognized that selecting appropriate management practices at activity venues assisted horses in adapting to changes to their daily routine and maintaining positive welfare states. The results will contribute to knowledge about the horse management, training, and performance practices undertaken by participants at activity venues and stakeholder perspectives about horse welfare and wellbeing.

## 2. Materials and Methods

### 2.1. Study Description and Sampling Frame

**Figure 1:**
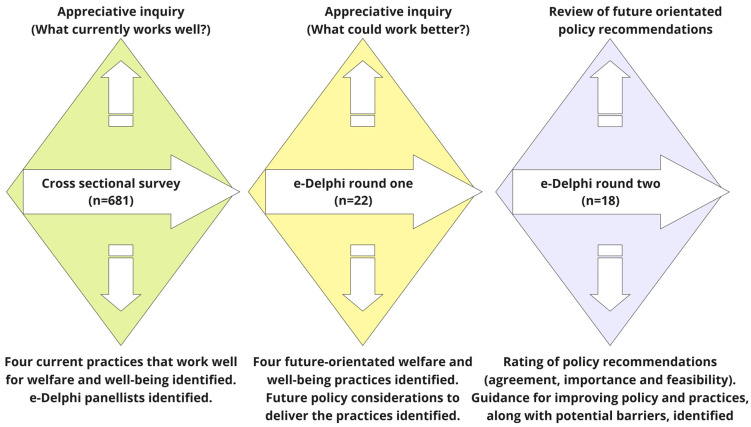
The Futurehorse project is a mixed-method study investigating horse sector participants’ attitudes about future-oriented practices for horse welfare and wellbeing. The study utilized three data collection methods: a cross-sectional survey and a two-round e-Delphi. Each diamond represents a data collection point. The cross-sectional survey contained six open-ended questions relating to practices for horse welfare outcomes, the results of which are reported in this study.

This study reports the analysis of a data subset from a larger interdisciplinary mixed-method study, titled the Futurehorse project, which investigated experienced horse sector participants’ perceptions of future-oriented practices for horse welfare and wellbeing. Data for the Futurehorse project were collected using an online cross-sectional survey and two e-Delphi rounds (Figure 1). A subset of the survey data, the responses to six open-ended questions relating to horse management practices at activity venues, is reported in this study.

The survey sampling frame required individuals to self-identify as having three or more years of involvement in a horse-related activity, as citizens of Australia or the United Kingdom (UK), as being over 18 years old, and as individuals or members of a team, involved with decision-making about horse, donkey, or mule welfare.

### 2.2. The Conceptual Framework

This study referred to the interpretivism perspective, recognizing that respondents’ attitudes toward horses, their experiences with them, and the performance of management practices are socially, culturally, and historically context-dependent [46,47]. The interpretivism theoretical perspective informed the selection of the appreciative inquiry method and the use of the Five Domains model as a tool to guide the research process [26,32,48]. The survey used an appreciative inquiry approach by asking respondents what practices they had experienced or witnessed being undertaken by organizers, venue managers, officials, participants, themselves, or other individuals at activity venues that they believed currently worked well for horse welfare [48]. The Five Domains model, which integrates the theoretical perspectives of biological function, affective state, and natural living, informed survey design, interpretation of results, and the discussion [26,49,50].

### 2.3. Survey Design and Distribution

The online survey collected data about demographic profiles using mixed response formats (open, closed, and multiple-choice questions) and about horse welfare-related practices using open-ended questions (refer to Supplementary Materials Item S1). Respondents were provided with pre-survey information regarding the Five Domains model, comprising a journal article and an infographic to improve accessibility [26,51,52]. Respondents were informed that reading the animal welfare information was optional. The six horse management practice-related survey questions were framed by four of the model’s domain headings: the physical environment, nutritional conditions, health, and behavioral interactions with (a) the physical environment, (b) other animals, and (c) humans [26]. As animal mental experiences are recognized within these four domains, the fifth domain, mental state, was not presented as a separate question in this study. A short explanation was provided for each question to increase the clarity, consistency, and reliability of responses [53]. Respondents were asked to think about one horse management practice at a time, mitigating risks for open-text responses becoming unwieldy and difficult to analyze. The draft survey was uploaded to Qualtrics [54] and tested by 27 volunteers, with feedback used to improve the final version. The survey link was promoted using the snowball method [55], with promotions including a radio interview, website articles, social media posts, and direct email contact with relevant horse-related organizations and businesses. The survey was conducted over 57 days between 5 July and 31 August 2021.

### 2.4. Analysis

Once the survey had closed, all responses were downloaded to Microsoft Excel [56] and de-identified by allocating a case number. Responses were eligible for analysis if at least one demographic question and one general horse-related question were answered, resulting in 57.2% (*n* = 681/1190) of responses deemed eligible for analysis [56]. Reasons for exclusion included failure to answer any questions, non-consent, or forced exit if self-assessed as non-citizens of Australia or the UK. Demographic data were prepared for analysis by categorization into country, age, gender, equid species of interest, amateur or professional status, job role, and years of experience within the sector. Data were described as the proportion (number and percentage) of respondents answering each of the six questions included in the current study. The denominator for each question varied due to non-responses.

**Figure 2:**
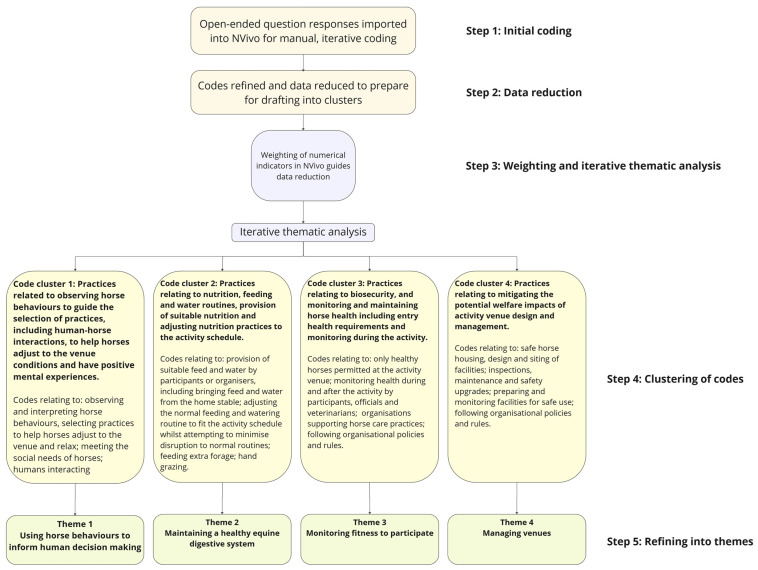
Coding methodology. Qualitative analysis followed a five-step process involving initial coding, data reduction, iterative thematic analysis, clustering of codes, and refining into themes.

Qualitative data analysis followed a five-step process (Figure 2), informed by Braun and Clarke [57]. Step 1 involved importing data into the NVivo [58] software application for management and manual, iterative coding techniques [57]. Initial coding rounds identified patterns and unified broad ideas and meanings (step 2). As the iterative coding process progressed, code refinement and data reduction occurred, guided by the weighting of numerical indicators within NVivo (step 3) [58]. These refined codes were clustered (step 4), informing the development of initial themes (step 5) [57]. All co-authors discussed the coding and thematic analysis periodically, adopting reflexive approaches by incorporating tools such as whiteboards to develop logic charts and critical feedback techniques [50,59]. The agreed themes synthesized the horse management practices respondents believed worked well at activity venues. As the iterative coding rounds used an inductive approach, the resulting four themes were not direct replications of the animal welfare domain that framed each survey question, and each theme could contain practice elements related to other themes. When reporting the results, parentheses containing case numbers were used to align results with the example illustrative quotes (refer to Supplementary Materials Item S2). The term ‘horses’ was used as a collective term that included mules and donkeys, reflecting that horses were reported as the most popular equid in this study.

## 3. Results

### 3.1. Demographic Results

Of the 681 respondents who provided survey responses eligible for analysis, the number answering each of the six open-ended survey questions varied. The largest number of responses was received for the question on horse health, the first question presented to respondents, and the smallest number for the question about behavioral interactions with other animals, which was the last question presented. The proportion of responses to each question was as follows: horse health (54.0%; 368), nutritional conditions (47.2%; 322), physical environment (44.6%; 304), behavioral interactions—humans (41.4%; 282), behavioral interactions—environment (38.4%; 262), and behavioral interactions—other animals (37.2%; 254). The demographic profile of respondents was similar for each of the six questions (Table 1).

### 3.2. Themes

#### 3.2.1. Theme One: Using Horse Behaviors to Inform Decision-Making

The theme Section 3.2.1 ‘*using horse behaviors to inform decision-making*’ identified horse management practices which, when undertaken at activity venues, were believed to work well to promote positive horse welfare and wellbeing. The results demonstrated that respondents interpreted horse behaviors and made inferences about how horses might feel to guide their selection of management practices and make decisions about appropriate interactions with horses, including when to provide them with undisturbed time.

Respondents regarded equine behaviors indicative of calmness as desirable because these were perceived as being safer for humans and other horses in proximity, and that horses might be experiencing a positive mental state (C 424,468,1169). They also believed that horses exhibiting calm behaviors were likely to perform better (C 874,971,60), bringing activity-related benefits in addition to equine welfare and wellbeing benefits. To inform the selection of management practices, respondents recognized that individuals needed to have the necessary skills to observe and interpret behaviors correctly and to handle and train horses respectfully, empathetically, and competently (C 301,527,936,968,1000). They also recognized that it was essential to monitor horse behaviors, as these might indicate horses were stressed for reasons such as changes to daily feeding routines and distractions, like show rides, spectators getting too close, or other animals, like dogs, chasing horses if not on a lead (C 60,47,468,779,951,985,1183). Practices that respondents believed worked well to help horses remain calm included arriving early at the activity venue and allowing enough time for hand-walking horses to let them explore their surroundings (C 763,821,874,1169). Hand walking was described as leading horses for exercise at the walk and, if permitted, eating grass (also referred to as hand-grazing or a grass pick) and being in proximity to other horses, which respondents perceived provided benefits for horse welfare and wellbeing (C 409,763,821,874,978,1073). Respondents recognized that horses needed to adjust to an activity venue’s housing conditions, including becoming accustomed to unfamiliar stables and yards or being tied up (C 60,737,867, 972,1008,1133). They also acknowledged that some practices involved trade-offs, for example, biosecurity-related practices were considered to promote horse health when done well but inevitably resulted in the horse’s social requirements not being fully met because, to manage disease risks, horses were kept apart (C 321,785,867,1133). However, respondents recognized that practices such as housing horses in proximity so they could see and hear each other, or arranging for a companion horse to be nearby, helped to meet social requirements and compensate for the effects of separation (C 218,785,796,837). Respondents also considered the importance of how humans interacted with horses and how people related and responded to each horse’s expressed behaviors. They acknowledged that the nature, manner, and frequency of human interactions with horses could influence the horse’s experience of the interaction and determine whether the overall experience was positive or negative (C 581,685,936,1005). For example, they considered that humans acting calmly around horses assisted horses in remaining calm. Practices like talking to horses and scratching them near the withers were also perceived as beneficial and as contributing to welfare and wellbeing (C 468,782,1005). Respondents also recognized that humans should demonstrate appropriate behaviors when training horses, describing empathetic and respectful attitudes as promoting positive experiences for horses. The selection of appropriate, evidence-based training practices incorporating consistent cues was also identified as likely to provide horse welfare and human safety benefits because horses exhibited more predictable behaviors (C 352,936,1074,1169). Overall, respondents believed that positive human attitudes and management practices that avoided adverse treatment of horses at activity venues were essential for promoting horse welfare and wellbeing. They also expected that officials at activity venues were empowered to intervene if these human behavior standards were not met (C 241,1146).

#### 3.2.2. Theme Two: Maintaining a Healthy Equine Digestive System

The theme Section 3.2.2 ‘*maintaining a healthy equine digestive system*’ describes practices that, when undertaken at activity venues, were believed to work well for preventing gastrointestinal diseases by adjusting and managing feeding regimes. These practices were perceived to benefit the horse’s physical health and mental experiences and, therefore, equine welfare and wellbeing. When relocating to an activity venue, respondents considered it essential to keep the horse’s dietary ingredients and feeding timetable as close as possible to those at the home stable because dietary changes might cause stress and increase the risk of gastrointestinal diseases (C 47,64,265,416,789). To mitigate these risks, and because venues did not usually provide feed, respondents explained that they brought pre-packed individualized diets, hay, and in some cases water to the activity venue (C 47,684,796,1073). There was an understanding among respondents that horse carers were responsible for each horse’s feeding and watering regime and deciding how to manage the horse’s nutritional requirements whilst accommodating the activity program requirements (C 658,265,789). Examples included feeding horses during breaks between activities, before horses leave the venue, or when they return to the home stable (C 241,265,303, 437,977). Respondents described practices they perceived as helping horses adapt to changed feeding conditions at the activity venue. These included feeding extra hay and using hay nets designed to slow down feed intake, perceiving that such practices extended the time horses spent eating, replicating natural foraging and grazing conditions, and could help reduce the occurrence of behaviors indicating negative mental states, such as boredom (C 64,265,416,454,789,973). Another practice, hand-grazing, was also believed to be beneficial for horses because they could eat and walk, and if allowed by handlers, to be near other horses (C 265,656,1056,1073).

Respondents acknowledged that individuals responsible for horses should possess sufficient knowledge about horses and equine nutrition for management practices to work well. They recognized that less experienced participants could seek advice from more experienced participants at the activity venue, identifying knowledge sharing as having potential welfare benefits (C 265,462). Respondents considered that officials checking horses were being fed and watered or responding to reports that horses needed to be checked contributed to horse welfare and wellbeing at activity venues (C 783,796).

#### 3.2.3. Theme Three: Monitoring Fitness to Participate

The theme Section 3.2.3 ‘*monitoring fitness to participate*’ identified practices related to monitoring and managing horses before, during, and after the horse-related activity that, when undertaken, were believed to work well for horse welfare and wellbeing.

Respondents recognized that only healthy horses in good body condition and with no visible signs of injury or disease should be brought to the venue and participate (C 489, 1003,1007). They acknowledged that officials could request veterinarians to examine horses and review documentation, like veterinary treatment or vaccination records, to provide advice on each horse’s health status and fitness to participate on arrival at the venue or any time during their stay (C 303,321,645,779,1007). However, not all organizations appointed veterinarians, instead relying on policies that empowered officials like judges or stewards to decide if horses were fit or unfit to participate (C 645,733). Respondents recognized that officials acted to protect horses by monitoring and enforcing compliance with the rules, such as mandated rest periods, medication control, and intervening if blood was visible on the horse (C 462,779,891,1146). Officials might also request an equine drug test or a veterinary examination to check a horse for signs of disease related to involvement in the activity, such as lameness, exercise-induced pulmonary hemorrhage, or evidence of improper equipment use or horse handling methods (C 261,658,779,891). In some circumstances, officials might determine that the horse is no longer fit to participate, resulting in the entry being withdrawn or eliminated, which respondents perceived to protect horses’ welfare and wellbeing (C 261,489,645,733,779).

Some management practices were selected in response to adverse horse health indicators, such as assisting hot horses in cooling down and recovering after exertion. Organizations supported such practices by providing ice buckets, misters, and fans at activity venues in hot weather and by appointing veterinarians who were also available for advice (C 267,923,974). Other practices were preventative, such as not allowing horses from different stables to mix or share equipment or feed, which acted to prevent injury and disease spread (C 321,737,1023). Respondents acknowledged that officials might decide to cancel or postpone activities in some adverse environmental conditions, such as extreme weather and heat, which they believed worked well to protect the welfare and wellbeing of horses and humans (C 698,1167).

#### 3.2.4. Theme Four: Managing Venues

The theme Section 3.2.4 ‘*managing venues*’ identified practices that respondents believed worked well to promote horse and human safety, health, and welfare when undertaken at the activity venue. These practices were related to venue preparation, management, and monitoring the venue once the activity had commenced. Preparation included inspection of the venue by officials to ensure that facilities and surroundings complied with the organization’s policies and that these were safe for humans and horses (C 466,658,925,1007,1133,1136). Respondents explained that appropriate inspections and monitoring included visiting facilities for housing horses, such as stables and yards, to check that these were well constructed, water was available, and bedding was provided if arranged. Inspections also included checking whether facilities like wash bays or services for waste removal had been arranged (C 619,658,867,881,925). In other situations, officials might check to ensure venue managers had provided enough space to tie up horses or to erect participants’ temporary yards if there were not enough permanent yards on site, another factor that helped keep horses safe from injury or disease (C 837,867,1133). Inspections needed to include performance areas to ensure that these were prepared before activities commenced and were monitored for the activity duration to ensure they remained safe to use (C 409,658, 705,790,925,1007). Respondents said that appropriate preparation and management of performance areas included practices like harrowing sand riding arenas, aerating grass racetracks, and undertaking upgrades like installing frangible technology on equestrian cross-country fences or plastic running rails at racetracks (C 403,455,555,705,925,977). They perceived these practices worked well because they improved overall safety for the horses and humans. A further venue management practice that was believed to work well when undertaken was monitoring human and horse traffic around entrances to performance areas and in warm-up arenas to avoid problems like overcrowding (C 60,218,737,892).

## 4. Discussion

This study investigated the opinions of experienced horse sector participants about practices undertaken at activity venues that they perceived worked well and contributed to horse welfare and wellbeing. The results showed that respondents recognized that relocating horses to activity venues created potential welfare challenges due to the changed living environment and routines the horses experienced. Taken as a whole, the practices described by respondents as working well to promote positive welfare and wellbeing served to assist horses in adapting to their new surroundings and changed routines and remain fit to participate. Analysis of qualitative data identified four themes relating to using horse behaviors to inform decision-making about practices and interacting with horses (Section 3.2.1. Theme One), maintaining a healthy equine digestive system (Section 3.2.2 Theme Two), monitoring horses for fitness to participate (Section 3.2.3 Theme Three), and managing the venue so that humans and horses remained safe (Section 3.2.4 Theme Four).

Respondents considered that a wide range of individuals at the activity venue were responsible for selecting appropriate management practices, including officials, veterinarians, and participants. The selection of appropriate practices extended to venue design and management, the provision of safe horse accommodation and performance areas, and facilities to help horses recover after the activity. Respondents felt that everyone interacting with horses had a responsibility to adopt empathetic and respectful behaviors toward horses. Respondents also considered it important that all activity participants and officials were able to correctly interpret equine behaviors in order to determine whether management practices resulted in positive mental experiences for the horse. While some practices were considered in terms of inputs needed to meet the horse’s requirements, such as provision of feed and water, others were considered more holistically, taking into account both inputs and outcomes or impacts that incorporated the horse’s physical health and mental experiences. This suggested that while some practices were aligned with the Five Freedoms model, others were aligned with animal-centric welfare frameworks like the Five Domains model. This suggests that a shift in attitudes to horse welfare and wellbeing is underway in the horse sector and that initiatives to implement the Five Domains model and update practices are achievable [15].

Respondents interpreted horse behaviors and made inferences about the horses’ mental experiences, using these evaluations to inform the selection of practices (Section 3.2.1 Theme One). Behavioral interpretations were also used to guide decision-making about appropriate interactions with horses, including when and how to interact as well as when not to interact with them for reasons such as allowing horses to rest. These findings are consistent with other studies reporting horse carers’ perspectives about equine behavioral interpretations and using these to inform the selection of practices, such as observing their horse’s reactions to noise and feeding more hay to help them relax [21,27].

Respondents explained how activity participants sought to assist horses to adapt to the venue environment and remain calm, perceiving that this provided health, welfare, wellbeing, and safety-related benefits for horses and humans. The finding resonates with other studies reporting that horses exposed to changed living environments, routines, or other unfamiliar or adverse situations can become stressed [3,4,22], and studies where horse behaviors indicative of calmness were considered safer for humans in proximity [4,23,60]. This study also found that participants might select practices like hand walking, perceiving that it assisted horses to become accustomed to their surroundings. Animals, including horses, benefit from opportunities to explore their surroundings, and experiencing repeated, managed exposure to stimuli can assist with habituation [39,61]. Respondents also acknowledged that practices involving humans interacting with horses should be informed by evidence-based approaches and undertaken by empathetic, skilled individuals who treated horses respectfully. They also recognized that organizations had a role to monitor interactions by appointing officials to enforce policies and rules that set expectations for human behaviors around horses. The findings suggest that organizations whose governance arrangements include officials and participants being held accountable for their actions are potentially in a position to provide horses with a robust welfare and wellbeing safety net [62,63,64].

Management practices designed to align nutritional regimes and routines at the activity venue with those at the home stable were perceived to work well (Section 3.2.2 Theme Two). The results showed that respondents recognized that nutritional practices needed to be selected that minimized the impacts of disruptions to the horse’s regular feeding regime whilst at the activity venue. Respondents understood that minimizing stress or disruption to nutritional regimes by providing extra hay and access to grass reduced the risk of gastrointestinal diseases, such as colic. These findings align with studies reporting that irregular feeding times can result in horses expressing behaviors indicative of negative mental experiences, and that feeding grass and hay to horses reduces risks for gastrointestinal diseases, such as colic [22,65,66,67]. Referring to the Five Domains model, confining horses at activity venues could result in a negative welfare state if, for example, access to food is unpredictable and the voluntary quenching of thirst is impeded [26,68]. This study’s results showed that while feeding and watering practices were perceived to work well overall, irregular feeding and watering regimes were believed by respondents to be an acceptable trade-off for human participation in activities. Although respondents recognized that horse carers were responsible for ensuring adequate feed and water provision for horses, the authors of this study propose that horse organizations also have an ethical obligation to ensure that horse activity programs minimize the frequency and duration of disruption to horses’ nutritional regimes that might result in situations where feed or water intake is reduced or withheld [19]. Further research is required to investigate how activity programming might better support equine feeding and watering regimes and minimize negative welfare-related impacts for horses.

Practices that monitored horses’ fitness to participate, when undertaken, were believed to work well to provide for horse welfare and wellbeing (Section 3.2.3 Theme Three). Respondents acknowledged that officials worked with participants to keep all horses healthy and to reduce the occurrence of injury and the potential for disease spread. Respondents also considered it was important that officials were empowered to monitor and enforce rules, including checking equine identification and veterinary treatment documentation, and arranging mandatory veterinary examinations. These findings align with studies reporting the use of veterinary examinations to diagnose issues relevant to activity participation, like lameness, to inform an official’s decision-making about a horse’s fitness to participate [44,69].

Venue management practices that provided the conditions for horse and human safety, welfare, and wellbeing were perceived to work well (Section 3.2.4 Theme Four). Respondents considered diverse aspects of managing horse activity venues, such as providing safe equine facilities, space to park equine transport, and resources to support management practices, such as water for cooling horses after exercise. These results aligned with white and grey literature about inspections, preparation, and ongoing monitoring of facilities at venues where humans and horses interact [23,43,70]. However, current activity venue inspection checklists are primarily human-centric [23,70,71,72] and do not combine the safety, welfare, and wellbeing requirements of humans, horses, and other species that may be involved in activities or are kept at the same location. When considering venue facilities, it was noteworthy that respondents did not describe design features in terms of appropriate enrichment levels or opportunities for expressing agency [16,39]. In contrast, community dog park design is evolving to include exploratory and sniffing experiences, demonstrating that it is possible to design animal facilities that are both functional and promote positive mental experiences for the target species [73,74].

Another aspect of venue management practices related to how facilities can support or inhibit the implementation of organizational biosecurity plans and practices. Past studies report that venues should have the capacity to quarantine horses for health checks before entering the main venue and for establishing isolation facilities should a suspect horse be identified [38,75,76]. While respondents in this study perceived that practices supporting biosecurity and horse health worked well, the co-authors noted that the health status of all horses at the activity venue could be compromised by venue design or management practices that allowed interaction of horses or activities where pre-activity health checks by veterinarians were not required. The literature reports how horse carers have disparate attitudes toward biosecurity and are not always motivated to undertake preventative practices consistently [77,78,79]. Studies about horse carers in Britain [79,80] and Australia [78,81] found that cultural and social factors influenced perceptions of disease risks and the effectiveness of preventative practices. Therefore, establishing a systematic approach to equine health that engages horse activity participants should be employed wherever horses from different home stables gather [38,76,82,83]. The current study found that while venue management practices were perceived to work well, the co-authors considered that the results indicated that venue practices currently favored human-centric goals, prioritizing the delivery of the activity and housing horses for this purpose. Further research is required to review venue design and management regimes using an animal-centric instrument like the Five Domains model and propose solutions to improve the life of the sentient horses present on the site, such as requirements for behavioral interactions and agency [16].

This study’s authors propose that while some management practices are described as inputs only, aligning with the Five Freedoms and provisions framework, other practices also consider outcomes, aligning with the Five Domains model. The findings suggested that activity participants may be receptive toward participatory organizational initiatives to implement the Five Domains model and update current practices accordingly. An example initiative is the Zoos SA’s Charter, which applies the Five Domains model in an animal care setting [84]. The findings of this study could be used by organizations to inform a start-up agenda for participatory approaches, such as a Community of Practice (CoP), designed to engage participants in finding solutions to improve horse welfare and wellbeing [85].

### Limitations and Future Research

This study is qualitative and inherently subjective; therefore, readers should read and interpret the results cautiously [57]. Data were collected using an online cross-sectional survey, a method that is subject to a range of biases [55]. These include recall bias, where the recall of information by survey respondents was influenced by factors such as previous experience caring for horses and the period lapsed since horses were last cared for [55,86]. Risks for recall bias were reduced because survey respondents self-assessed as having current involvement in the horse sector and having three or more years of experience. Confirmation bias, in which individuals tend to interpret information in alignment with their beliefs, was also a potential limitation. Framing questions using the headings from the Five Domains model and providing access to an article and infographic on the model assisted in reducing, but not eliminating, risks associated with this bias. Respondents were not asked questions related directly to the fifth domain of the Five Domains model, mental experiences, which could be considered when planning future research projects. Further research is required to define the extent to which current horse practices undertaken at activity venues provide opportunities for horses to experience practice outcomes positively [26].

## 5. Conclusions

This study inquired about the perspectives of experienced horse sector participants regarding which practices undertaken at horse activity venues they perceived worked well for horse welfare and wellbeing. The findings provided insights into the complexities of managing horses relocated to activity venues and the importance of using behavioral interpretations to make decisions and adapt practices to assist horses to remain calm, healthy, and fit to participate. This study’s findings suggest that activity participants may be receptive to updating current practices and referring to the Five Domains model when evaluating the benefits of practice outcomes for horses. The findings also suggest that there is scope to improve current practices, for example, by understanding factors that impact decision-making about restricting or withholding feed and water. Updating practices continuously for the benefit of horses is likely to contribute to their safeguarding and, more broadly, maintain the sector’s social license to operate.

## Figures and Tables

**Table 1:** The demographic characteristics of participants who were eligible for inclusion in the Futurehorse survey (*n* = 681), stratified by the question(s) answered. Data were collected between 5 July and 31 August 2021.

		Question					
Demographic	Category	Horse Health *n* (%)	Environment *n* (%)	Nutrition *n* (%)	Behaviour Human *n* (%)	Behaviour Environment *n* (%)	Behaviour Other Species *n* (%)
Respondents	Amateur	194 (56.1)	165 (57.9)	173 (57.7)	155 (59.2)	141 (58.0)	138 (58.2)
	Professional	152 (43.9)	120 (42.1)	127 (42.3)	107 (40.8)	102 (42.0)	99 (41.8)
Job role	Activity manager	162 (48.9)	133 (47.2)	143 (48.3)	126 (48.6)	120 (49.4)	113 (47.7)
	Horse management or training	44 (13.3)	37 (13.1)	38 (12.8)	32 (12.4)	30 (12.3)	27 (11.4)
	Role not specified	24 (7.3)	21 (7.4)	23 (7.8)	19 (7.3)	19 (7.8)	20 (8.4)
	Participant	17 (5.1)	15 (5.3)	15 (5.1)	14 (5.4)	14 (5.8)	14 (5.9)
	Vet services	21 (6.3)	18 (6.4)	17 (5.7)	15 (5.8)	13 (5.3)	15 (6.3)
	Welfare, rescue, retraining	9 (2.7)	8 (2.8)	8 (2.7)	6 (2.3)	6 (2.5)	7 (3.0)
	Other job role *	54 (16.3)	50 (17.7)	52 (17.6)	47 (18.1)	41 (16.9)	41 (17.3)
Activity	Equestrian sports	165 (49)	136 (48.7)	145 (49.2)	128 (50.0)	115 (48.3)	111 (48.3)
	Other horse sports	37 (11.0)	31 (11.1)	33 (11.2)	27 (10.5)	27 (11.3)	28 (12.2)
	Horse racing	44 (13.1)	33 (11.8)	33 (11.2)	30 (11.7)	28 (11.8)	27 (11.7)
	Multiple activities	21 (6.2)	17 (6.1)	18 (6.1)	14 (5.5)	15 (6.3)	12 (5.2)
	Recreation **	36 (10.7)	32 (11.5)	34 (11.5)	29 (11.3)	25 (10.5)	26 (11.3)
	Breeding	3 (0.9)	2 (0.7)	2 (0.7)	2 (0.8)	2 (0.8)	2 (0.9)
	Other types of activity ***	31 (9.2)	28 (10)	30 (10.2)	26 (10.2)	26 (10.9)	24 (10.4)
Species	Horse	358 (98.1)	269 (97.8)	314 (98.1)	274 (97.9)	255 (97.7)	247 (97.6)
	Horse, donkey, and/or mule	4 (1.1)	4 (1.5)	4 (1.3)	4 (1.4)	4 (1.5)	4 (1.6)
	Donkey	3 (0.8)	2 (0.7)	2 (0.6)	2 (0.7)	2 (0.8)	2 (0.8)
Gender	Female	312 (85.7)	260 (86.1)	277 (86.6)	241 (86.1)	223 (85.8)	216 (85.7)
	Male	52 (13.5)	42 (13.2)	43 (13.4)	39 (13.9)	37 (14.2)	36 (14.3)
Citizen	Australia	334 (90.8)	277 (91.1)	291 (90.4)	257 (91.1)	240 (91.6)	232 (91.3)
	United Kingdom	34 (9.2)	27 (8.9)	31 (9.6)	25 (8.9)	22 (8.4)	22 (8.7)

* Other job role includes other roles with limited numbers combined, e.g., media officer ** Recreation includes leisure e.g., trail riding, packing, driving. *** Other types of activity includes other activities with limited numbers combined, e.g., welfare, rescue, and retraining.

## Data Availability

The data supporting this study’s findings are not publicly available due to privacy or ethical restrictions.

## Supplementary Materials

The following supporting information can be downloaded at: https://www.mdpi.com/article/10.3390/ani15152182/s1. Supplementary Document S1: Horse activity participants’ perceptions about practices undertaken at activity venues, and horse welfare and wellbeing. Supplementary Document S2: Horse activity participants’ perceptions about practices undertaken at activity venues, and horse welfare and wellbeing.
