# Supplementary material for: Horse Activity Participants’ Perceptions About Practices Undertaken at Activity Venues, and Horse Welfare and Wellbeing
Source: Animals (Basel). 2025 Jul 24;15(15):2182. doi: 10.3390/ani15152182 (PMC12345512; doi:10.3390/ani15152182)
Supplement: Supplementary file 1 [file animals-15-02182-s001.zip › Supplementary Information Item S1.pdf]

Horse activity participants' perceptions about practices undertaken at activity venues, and horse welfare and wellbeing.

Supplementary Information Item S1: Cross-sectional survey: the six questions reported in this study.

https://doi.org/**10.5281/zenodo.15787355**

1: Topic: Horse health
Preamble: This section relates to horse health while at the activity location, for example, a racecourse, showground or a mountain trail. This section refers to topics such as fitness levels, injury, disease and physical condition. We are interested in current practices, or 'ways of doing things', which contribute towards horse welfare.
Question: Provide one example of current practice which you consider is done well. The practice is in the context of horse health at the location for your selected activity.

2: Topic: Nutritional conditions
Preamble: This section relates to the nutritional conditions while at the activity location, for example, showground or racecourse. This section refers to topics such as the quality and quantity of water, and the quality, quantity and varieties of food. We are interested in current practices, or 'ways of doing things', which contribute towards horse welfare.
Question: Provide one example of a current practice which you consider is done well. The practice is in the context of nutritional conditions while at the location for your selected activity.

3: Topic: The physical environment
Preamble: This section is about the physical environment at the location where your selected activity takes place, for example, beach, riding arena or racecourse. This section refers to topics such as built infrastructure, horse accommodation and aspects related to the natural environments, for example, air quality, noise level, lighting, surface conditions and physical limits on allocated space per horse. We are interested in current practices, or 'ways of doing things', which contribute towards horse welfare.
Question: Provide one example of a current practice which you consider is done well. The practice is in the context of the physical environment while at the location for your selected activity.

4: Topic: Horse behaviours- interactions with people
Preamble: This section is about horse behaviours and interactions with people while horses are at the location where your selected horse activity takes place, for example, trotting track, indoor riding arena or national park trails. This section refers to topics such as human attitudes or handling, riding or driving skills. We are interested in current practices, or 'ways of doing things', which contribute towards horse welfare. The experience for the horse is relational to the conditions or circumstances available for interaction.
Question: Provide one example of a current practice which you consider is done well at the location where the activity takes place.

5: Topic: Horse behaviour - interactions with the environment
Preamble: This section is about horse behaviour and interactions with the environment while the horse is at the location where your selected horse activity takes place, for example, racecourse, tourist destination or showground. This section refers to topics such as foraging, exploring and movement. We are interested in current practices, or 'ways of doing things', which contribute towards horse welfare.
Question: Provide one example of a current practice which you consider is done well at the location where the activity takes place.

6: Topic: Horse behaviours - interactions with other horses, or other animals
Preamble: This section is about horse behaviours and interactions with other horses, or other animals while the horse is at the location where your selected activity takes place. This section refers to topics such as bonding, playing, retreat and rest. We are interested in current practices, or 'ways of doing things', which contribute towards horse welfare.
Question: Provide one example of a current practice which you consider is done well at the location where the activity takes place.
