# Supplementary material for: Horse Activity Participants’ Perceptions About Practices Undertaken at Activity Venues, and Horse Welfare and Wellbeing
Source: Animals (Basel). 2025 Jul 24;15(15):2182. doi: 10.3390/ani15152182 (PMC12345512; doi:10.3390/ani15152182)
Supplement: Supplementary file 1 [file animals-15-02182-s001.zip › Supplementary Information Item S2.pdf]

Horse activity participants' perceptions about practices undertaken at activity venues, and horse welfare and wellbeing.
Supplementary Information Item S2 Example illustrative quotes
https://doi.org/10.5281/zenodo.15787729

Case No.	Example illustrative quotes
47	'Horses are fed both water and feed from home to ensure they are comfortable and do not fall ill from a change in diet or routine.'
60	'Horses can get very excited at the races so race clubs exclude the general public from getting too close to the horses so they can relax a bit pre and post-race protects the horses and the people.'
60	'At the races, the horses are kept in stalls where they can see other horses but not physically touch for horse health reasons, spreading disease etc.'
64	'Keeping the feeding at routine times and keeping the diet as close to natural nourishment as possible with plenty of roughage included and continual access to clean water.'
93	'Not overcrowded with people [or] horses, good space to ride and work.'
218	'Horses stabled or yarded close to each other so social needs are met.'
241	'Kids are encouraged and reminded to feed and water their ponies at break times.'
241	'There are set club days where a trained coach is present to give advice and teach. The most important factor in horse welfare is the owner [because] a better trained owner leads to better horse welfare outcomes.'
261	'Judges, stewards [and] officials always have the horses welfare as the priority, with all having the right to call for vet examination of a horse prior to allowing it to continue in the competition.'
265	'Horses often have [a] change in access to roughage at competitions…Horse owners and riders are very good at balancing and adjusting feeds and practices to accommodate this, for example, hand grazing, increasing hay offered, etc.'
267	'Keeping the horse fit and well is crucial in racing. The current practice here (identifying name removed) providing misters to the stalls and ice buckets in hot weather.'
301	'When in work both trainers and their staff handle my horses gently and competently, firm when required but never roughly.'
303	'Horses are continually being scrutinised for evidence of anything which may be affecting their performance by stewards and on course veterinarians and if there is any doubt trainers are requested to have inspections with or without diagnostic testing with certification to ensure it is fit to race.'
303	Horses are rarely fed on racetracks and are usually offered water post-race.'
321	'Vaccinations are often checked by venues. We would be mindful of biosecurity if taking the horses out not mixing with other horses or sharing equipment, food, water etc., if mixing had to happen, e.g. when some of the liveries have stabled elsewhere for camp for a few days they are quarantined on return.'
352	'Horses have been trained in hand and under saddle and are easy to handle and predictable in their behaviours'
403	'Trained and experienced course designers are responsible for cross country fence design and build every effort is made to ensure the safety of horses and riders with the design of fences and with the use of frangible technology.'
409	'Keeping arena surfaces from being too dusty, especially important for indoor venues.'
409	'Horse owners sometimes hand graze their horses for short periods to get the[m] out of the stables.'
416	'Trying to mimic natural horse foraging by providing large slow feeder hay nets filled with grass hay day and night. The practice helps prevent stress, boredom, [and] equine gastric ulcer syndrome (EGUS).'
424	'I am also encouraging and teaching others how to apply these [equitation science] principles which is of benefit to the horses and also making the hospital environment and interaction with horses safer and more predictable for horses and humans.'
437	'Monitoring of hydration and metabolic condition.'
454	'Horses are provided with haynets, [hay] bags and fresh water while not taking part in activities.'
455	'Riding arena is harrowed before each event day.'
462	The ability to get expert advice and assistance from highly knowledgeable nutrition specialists available.'
462	The practice of governance over aids is a good example spur lengths whip usage and noseband fits are all to ensure comfort and safety for both horses and rider.'
466	'We always do a risk assessment to consider the horse and other people.'
468	'The horses are always managed quietly calmly with respect we are privileged to be working with such amazing athletes horses thrive on routine so we do everything the same at competition as at home.'
489	'All horses are in good condition. No horse can compete if there is any sign of injury blood lameness.'
527	'Horses are treated with respect at all times [and] all staff are suitably trained and supervised whilst handling horses.'
555	'Preparation of consistent track surfaces and cambered tracks have reduces musculoskeletal injuries when racing.'
581	'Horses behaviour is bought to the attention of the client so they can recognise and respond to the horse communicating their needs such as walking away moving away, tail swish, ears back.'
619	Adequate water points and wash bays.'
645	'In dressage a judge will disqualify a lame horse or one which they see as a welfare issue. In eventing the horses are vetted after the cross country, in endurance the horses are vetted pre-ride [and] at the end, and depending on the length of the ride, at various points on track. Horse welfare is a major concern in all three.'
656	'Clean water is always available, and at events, horses are confined to a smaller yard; therefore are given supplementary feed and hand walked to graze at regular intervals.'
658	'Our sport rules dictate what must be provided for horses at events. Stables, yards are the correct size, sufficient bedding, water available and all spoiled bedding removed daily, and riders [and] those responsible monitor condition daily, and [the] area stewarded regularly for safety and surrounds.'
658	'Horse inspections prior to competitions and observations of sport. Stewards drug testing.'
684	'Gut health is considered through adequate access to feed as well as high-quality drinking water and supplements to support performance and recovery.'
685	'The horse is treated as an individual and his personal idiosyncrasies taken into account.'
698	'A hot weather policy protects the welfare of horse and rider.'
705	'Arena surfaces are graded and monitored for competition quality and reduce potential strains, injuries to horses.'
733	'I am limited to small shows where there is no official vet presence; however, if a horse has a health problem, most commonly lameness, a judge or other official will talk to the owner and the horse will be withdrawn.'
737	'Horses and pedestrians kept separate.'
737	Horse[s] are restricted from nose-to-nose contact and sharing feed and equipment due to biosecurity policies.'
763	'Horses are able to be taken for walks or [a] pick [of grass] or stabled if horses settle better.'
779	'Health examinations by testing veterinarians, the veterinary delegate, the ground jury, technical delegate and assistant technical delegate. Stewards observe the stable area to watch that the horses are well cared for. At international events they are rostered on day or and night, wristbands and identification are checked for anyone coming in and out of the stables [and] horses must wear identification numbers at all time[s]. The stables are securely protected by a huge cyclone fence no identification means strictly no entry hence tampering with horses is closely monitored. Stewards supervise all warming up arenas, the ground jury eliminate any lame horses, treatment documents are given to the veterinarians who supervise this or prohibit any
779	'Dogs must be kept on leads, hence no chasing or scaring of horses, [dog] fights or bitten spectators.'
782	'At an event, we will spend more time with the horse walking, grooming [and] even sitting outside the stable to be there to talk and interact.'
783	'As a steward, we check stabling areas to ensure that horses have fresh water, adequate bedding and provided with quality feed.'
785	'This [horse to horse interactions] is not encouraged at events due to biosecurity reasons; however, you will see horses that have arrived together are allowed to bond and scratch each other.'
789	'For this subject nutrition is the responsibility of the owner. The horses would be fed the same at a show or have more feed as they would be yarded or stabled.'

790 ‘Arena surface conditions are constantly monitored by stewards, judges and competitors.'

796 ‘All equestrian events, either national and or international qualifiers, require organising committees to predetermine access for riders and their horses to adequate and uncontaminated water.
Food supplies for the horses are not part of the event organiser's role; however, if it was reported that a horse was not being adequately provided for this would require further investigation by an official and a veterinarian if applicable.’

796 ‘Equestrian as a sport does not tolerate poor human attitudes or handling of horses … some disciplines specifically issue warning cards for poor human behaviours or attitudes to horses.'

796 ‘At equestrian events or training activities the primary purpose of the human horse interaction is participation in an equestrian-related activity, not for horse social bonding, retreat or rest activities.
However, housing horses with a visual view of other horses promotes social connections.
Providing horses with rest periods between activities, event or training scheduling promotes best practice horse care and welfare.’

796 ‘Generally, all equestrian event[s] and activities require an inspection of the grounds and facilities, many of which are documented and require completion prior to any equestrian activity.
Officials such as course designers, obstacle builders and technical delegates form part of the review team for eventing, and they are responsible for checking the course is as safe as reasonably practical and meet relevant rules and guidelines.
Adequate accommodation, access egress, arena course surfaces and environmental conditions are the responsibility of the organising committee, coach or affiliated club each state.'

821 ‘Arriving early and allowing the horse to settle in the different environment before competing.’

837 'Temporary holding for overnight competitions is regulated within the rules to ensure safety.'

837 ‘Rules dictate that a horse may have a companion attend with him her at veterinary checks as we understand they can become anxious if separated from their mate.’

867 ‘The stables are of a robust construction and are well maintained as this is a regulated sport.'

867 ‘Horses at the races are housed individually in a way that limits horse to horse contact and therefore minimises the chances for disease spread between stables of origin.'

874 ‘Horses remain calm despite sudden external stimuli e.g. having a pick of grass and can remain calm with a truck driving by.’

881 ‘Adequate facilities to exercise, wash down, sand roll or put horses on walker.'

882 ‘Stable complexes are locked for periods at night to enable horses to rest and not be disturbed by grooms riders entering at all hours.’

891 ‘Elimination if blood on flanks in spur area, elimination on bloody nasal discharge.'

892 ‘[The] main competition area is fully fenced and can only be accessed by horses going through the gear check shoot [sic] onto the competition area that has three arenas.
Judges access the arena via a separate driveway, allowing a large distance between the two, thus lessening the chance of frightening the horses.'

923 ‘Some racecourses have misters and fans in the stalls for hot days.'

925 ‘Site inspections prior to the event, including the condition of the surface, stables, water quality, etc.’

936 ‘Whether or not the experience is a positive one for the horse can depend on the empathy of the individual presenting the horse in the main horse have been prepared for this experience and the timings of the day
and locations of events are established in order to make the experience as positive as possible.’

951 ‘Most owners keep close watch over their animals at competitions.’

968 ‘Good training helps horses make good decisions and responses in a competition environment.’

971 If you want a horse to perform at its best, they need to be relaxed and not under undue stress.'

972 ‘Horses are stabled next to each other where they are able to see each other; at some venues they are able to touch noses.’

973 ‘Riders are providing their horses with haynets, so they always have something in their stomachs in stressful environments.’

974 ‘Vet checks of horses post cross country with cooling areas well set and advice to competitors provided pre-competition.'

977 ‘One thing that has been great is the use of plastic running rails on racecourses. They have prevented a huge number of injuries to both horse and rider.'

977 ‘You don't feed a horse while it is at a racecourse before its race. They are fed in the morning before they leave and after they get home.’

978 ‘At dressage events, horses need to be penned or on lead with ID numbers on them at all times. If a horse is foraging, it is on lead. It cannot explore on its own.’

985 ‘At shows usually good efforts are made to ensure the show arena is horse friendly i.e., no large scary sideshows nearby.'

1000 ‘The vast majority have kind caring and well experienced handling of horses at events and it is very common for others to help out and advise others who may not be so knowledgeable.’

1003 ‘At showjumping events horses are in good physical condition healthy generally fit for purpose and free of injury and disease.'

1005 ‘Horses have positive interactions with people trainers based on positive reinforcement training methods and regular affiliative interactions such as wither scratching.’

1007 ‘We have minimal infrastructure and according to the base we will make sure that there is a solid and suitable area to veterinary inspect horses in and out on even surface to check for lameness.'

1007 We have veterinarians to ensure that we are looking after our horses properly, and they can educate [us] and thoroughly examine the horses before entering the competition, during, and at
the conclusion, to ensure that the horse is fit enough, and it has been managed appropriately.'

1008 ‘Horses are kept in separate yards or tied up to floats; there is not a lot of horse interaction.’

1023 Not allowing horses to drink out of troughs on site water decanted into owners own bucket for use to help stop the spread of diseases.'

1056 ‘Horses are enriched throughout the day by human and other horse interactions, grazing, hand walking feeding, grooming etc.’

1073 ‘We prepack each meal for each horse prior to travel and sometimes carry our own water for horses with fussy palate.'

1073 ‘I think we do this as well as one can but it it is not ideal for horses to be stables and away from their mates and constantly controlled i.e., on lunge or lead or under saddle our horses seem content enough with the practices we use feeding damp hay regularly,
cleaning stables regularly, walking [and] taking them for a [grass] pick with their mates etc.'

1074 ‘The riding and training of the horse when out employs the use of both positive and negative reinforcement this provides the horses with a consistency which reduces their stress as much as possible.’

1133 ‘Yards and sufficient area around floats for safe tying prevents horse-to-horse interaction, preventing injury and potential for infection.'

1136 ‘Ensuring the location grounds is suitable to compete on and not during adverse weather conditions…committees must comply providing fresh water collection of manure grounds are suitable to ride on surfaces etc.'

1146 ‘Stewards monitoring warmup and housing areas to make sure that abuse during training sessions do not occur.’

1146 ‘Rules that enforce that horses have quiet time after cross country between certain night time hours and stewards are in place to make sure that horses get this rest and are not exposed to prohibited medications practices etc.’

1167 ‘For extreme weather, hot or cold, [or] wet, our competitions are generally postponed or cancelled.'

1169 ‘Spending time allow horses to explore their environment prior to being expected to perform is critical for safe events and relaxed horses.'

1169 'Most of our members have some understanding of learning theory horse behaviour and using positive reinforcement a small component do not and we work
to educate those members and bring them up to date on modern training methods with better welfare outcomes'.

1183 ‘All care is taken with environmental situations at shows. most of the time organisers try to have the environment safe and happy for horses but in saying that at agricultural often outside influences
like rides sideshow noise etc. affect a horses behaviour.’

Note: [] Square bracketing indicates text inserted to improve readability.
